# Randomized Clinical Trial of Four Adhesion Strategies in Non-Carious Cervical Lesion Restorations: Five-Year Follow-Up

**DOI:** 10.3290/j.jad.c_2318

**Published:** 2025-10-30

**Authors:** Érika Mayumi Omoto, Caio César Pavani, Paulo Roberto Marão de Andrade Carvalho, Mirela Sanae Shinohara, Bruna Perazza, Ticiane Cestari Fagundes

**Affiliations:** a Érika Mayumi Omoto PhD student, Department of Restorative Dentistry, School of Dentistry, Araçatuba, SP, Brazil. São Paulo State University (UNESP). Literature investigation and writing the manuscript.; b Caio César Pavani Assistant Professor of Restorative Dentistry, Department of Restorative Dentistry, School of Dentistry, São Paulo State University (UNESP), Araçatuba, SP, Brazil. Manuscript writing and review.; c Paulo Roberto Marão de Andrade Carvalho PhD, Department of Restorative Dentistry, School of Dentistry, Araçatuba, São Paulo State University (UNESP), SP, Brazil. Literature investigation, experimental design, methodology performance, and data curation.; d Mirela Sanae Shinohara PhD, Department of Restorative Dentistry, School of Dentistry, Araçatuba, São Paulo State University (UNESP), SP, Brazil. Literature investigation, experimental design, methodology performance, and data curation.; e Bruna Perazza MS student, Department of Restorative Dentistry, School of Dentistry, Araçatuba, São Paulo State University (UNESP), SP, Brazil. Literature investigation and manuscript writing.; f Ticiane Cestari Fagundes PhD, Associate Professor of Restorative Dentistry. Department of Restorative Dentistry, School of Dentistry, Araçatuba, São Paulo State University (UNESP), SP, Brazil. Idea and hypothesis conceptualization, project administration, supervision, and manuscript review.

**Keywords:** acid etching, adhesive system, dentin, randomized controlled clinical trial, composite resin, resin-modified glass-ionomer cements

## Abstract

Several adhesion strategies have been proposed for restoring non-carious cervical lesions (NCCLs). Long-term clinical trials are essential to guide clinicians toward the most effective approach.

**Purpose:**

To evaluate the 5-year clinical performance of NCCLs restored using different adhesion strategies.

**Materials and Methods:**

Four strategies were assessed: an adhesive system (Scotchbond Universal; SBU/Filtek Z350XT) applied without (SBU) and with selective enamel conditioning (E-SBU), and a resin-modified glass-ionomer cement (Vitremer; RMGIC) applied without (RMGIC) and with ethylene-diaminetetraacetic acid pretreatment (E-RMGIC). Two hundred restorations were evaluated using United States Public Health Service criteria after 5 years. Kappa test, Wilcoxon Signed-Rank test, Kaplan–Meier analysis, equality tests of two proportions, and multiple logistic regression were applied (α = 0.05).

**Results:**

One hundred and nineteen restorations were re-evaluated after 5 years. No significant differences were observed in restoration survival among groups. E-SBU exhibited more Bravo scores for marginal integrity compared to the ionomer groups and for marginal discoloration compared to RMGIC. Ionomer groups had higher Bravo scores for surface texture. RMGIC showed greater wear than E-SBU, being statistically similar to SBU. No significant differences were found for color and secondary caries over time. Multiple logistic regression analysis revealed that restoration retention was significantly influenced by the type of tooth (premolars), the degree of dentin sclerosis, and the degree of marginal tissue recession.

**Conclusions:**

All four adhesion strategies demonstrated similar 5-year survival. Marginal defects were more frequent in the selective enamel etching group than in ionomeric restorations. Surface luster was reduced in ionomer restorations. The retention of NCCL restorations may be affected by tooth type, degree of dentin sclerosis, and marginal tissue recession. The four adhesion strategies had similar survival after 5 years; however, composite resin restorations applied after selective enamel etching promote more initial marginal defects than ionomer-based restorations following the manufacturer’s instructions. Clinical factors such as lesion location, dentin sclerosis, and gingival recession can influence the retention of NCCL restorations.

In clinical studies in Restorative Dentistry, trials involving non-carious cervical lesions (NCCLs) are particularly important due to their high prevalence in the population.^52^ Furthermore, these lesions are ideal for evaluating adhesion to dental substrates due to their anatomical configuration.^6^ The main etiological factors associated with these lesions include: excessive consumption of acidic and carbonated beverages (71.4%), gastroesophageal reflux disease (14.3%), improper brushing technique (28.6%), habits such as nail biting (14.3%), daily consumption of sunflower seeds (9.5%), use of toothpicks as a hygiene aid (19.0%), and the parafunctional habit of tooth grinding (4.8%).^45^

Advancements in adhesive systems have led to the development of several strategies for restoring NCCLs.^9^ The fundamental principle of adhesion to dental structures involves the demineralization of the substrate, followed by infiltration with resin monomers.^6^ The chemical bonding potential of functional monomers in adhesive systems and in ionomeric cements has been widely reported in the literature, particularly for its role in enhancing long-term adhesive durability.^29,48^

Universal adhesives were introduced to the dental market about a decade ago.^21^ These versatile materials can be applied using both etch-and-rinse and self-etch techniques.^21,27,37^ Phosphoric acid etching of dentin removes the smear layer and demineralizes the substrate, exposing collagen fibers that are subsequently infiltrated by resin monomers to form a hybrid layer.^6^ However, if the depth of dentin demineralization exceeds the infiltration capacity of resin monomers, hybrid layer formation may fail, leaving deeper collagen fibrils exposed.^22^ Exposure of collagen fibrils, combined with reduced pH and dissolution of calcium phosphate, can activate previously trapped components in the substrate, such as matrix metalloproteinases (MMPs).^22^ Since phosphoric acid conditioning involves dentin demineralization, the release of these enzymes is inevitable.^22^ In view of these consequeces, universal adhesive systems are primarily based on the chemical interaction between bifunctional acidic monomers and hydroxyapatite in the dental substrate.^59^ This interaction enables the formation of a stable chemical bond that is less susceptible to hydrolytic degradation.^59^

In this context, glass-ionomer cements are a viable option for restoring NCCLs, as they do not expose collagen fibrils, adhere chemically to the tooth structure, and exhibit a coefficient of thermal expansion similar to that of dentin.^48^ Resin-modified glass-ionomer cements (RMGICs) contain the same essential components as conventional glass-ionomers – basic glass powder, water, and polyacid – but also include the monomer 2-hydroxyethyl methacrylate (HEMA) and a photoinitiator.^32^ Using ethylenediaminetetraacetic acid (EDTA) before placing glass-ionomer cement may serve as an alternative preconditioning method.^11^ It dissolves the mineral phase of dentin without altering its organic matrix, producing moderate demineralization that can facilitate adhesion to the residual mineral within collagen fibrils.^11^ *In-vitro* studies that employed EDTA as a dentin pretreatment prior to the placement of RMGIC demonstrated satisfactory bond strength values.^1,11,15^ Additionally, EDTA has been shown to inhibit MMPs.^15^ Although EDTA has shown promising *in-vitro* results,^1,11^ the current understanding of its clinical effectiveness is still insufficient,^15^ emphasizing the need for further long-term follow-up clinical trials to support its application in dental practice.

The performance of NCCL restorations using composite resins or glass-ionomer cements has shown conflicting results, largely due to the wide variety of adhesive strategies and commercial restorative materials.^18^ Despite this variability, both materials have demonstrated satisfactory short-term clinical outcomes in NCCL restorations.^14,20,25,43,47,62^ However, long-term follow-ups are necessary, mainly to evaluate recent adhesion strategies.^13,26,31,33,40,44^

The main outcome of the present study was to compare the 5-year survival analysis of different adhesive strategies in NCCLs. The secondary outcomes included identifying differences among adhesive strategies with respect to clinical parameters and assessing changes over time within each adhesive strategy. The influence of initial lesion characteristics on restoration retention was also investigated. Then, the null hypotheses tested were: (1) There would be no statistically significant difference in the survival analysis among the four adhesive strategies after 5 years; (2) There would be no statistically significant difference in clinical parameters among the four adhesive strategies after 5 years; (3) There would be no statistically significant difference between baseline and 5-year evaluation for the same adhesive strategy; (4) The initial characteristics of NCCLs would not influence the retention of the restorations after 5 years.

## MATERIALS AND METHODS

### Study Design, Ethics Approval, and Protocol Registration

This prospective, randomized, double-blind, split-mouth study aimed to evaluate the 5-year survival and clinical performance of NCCL restorations using two restorative materials with different adhesive strategies.

The following groups were established: SBU – Scotchbond Universal/ Filtek Z350XT (3M ESPE, St. Paul, USA) used without selective enamel conditioning; E-SBU – Scotchbond Universal/ Filtek Z350XT with selective enamel etching; RMGIC – resin-modified glass-ionomer cement (Vitremer; 3M ESPE, St. Paul, USA) used without pretreatment; E-RMGIC – with EDTA pretreatment clinical evaluations were performed at baseline and after 1, 2, 3, 4, and 5 years.

The study protocol and informed consent form were approved by the local ethics committee for human research (#668.963) nd registered in the Brazilian Registry of Clinical Trials (RBR-655c3z). All participants provided written informed consent prior to treatment.

### Sample Size and Patient Selection

Sample size calculation was based on the reported retention rate of Adper Single Bond (3M ESPE, St. Paul, USA), a simplified etch-and-rinse adhesive and predecessor of the multi-mode adhesive tested, which showed a retention rate of 94% at 18–24 months of follow-up.^22,40,34^ A minimum of 50 restorations per group was required to detect a 20% difference between groups, with 80% power and two-sided testing.^41^

Fifty volunteers from the local undergraduate clinic were included. Each participant was presented with at least four NCCLs, regardless of their location in the dental arch.

### Inclusion and Exclusion Criteria

Inclusion criteria included good general health, no known allergies to dental materials or medications, and adequate oral hygiene, defined as the absence of active periodontal disease. Exclusion criteria included pregnancy or nursing, presence of active carious lesions, use of desensitizing agents or fluoride, orthodontic appliance use, and severe bruxism with more than 50% tooth wear.

### Selection of Teeth

Selected teeth exhibited NCCLs with depths ranging from 1.0 mm to 3.0 mm and with at least 50% of the cavity margins lacking enamel. All teeth showed no signs of periapical alterations, confirmed by clinical examination including pulpal vitality testing. No attempt was made to determine the etiology of the cervical lesions.

### Previous Evaluation

Oral health conditions were assessed using the decayed, missing, and filled teeth index (DMFT), the visible plaque index (VPI), and the gingival bleeding index (GBI).

Initial characteristics of the teeth and cavities were recorded, including tooth type; cavity dimensions (height, width, and depth in millimeters); internal cavity angulation (categorized as 45–90°, 90–120°, or >120°); degree of dentin sclerosis (graded from 1 to 4); and the level of marginal tissue recession (classified as I to IV).

Dentin sclerosis was graded as follows^50^: 1 – No evident sclerosis: opaque dentin with a yellow or whitish appearance and minimal discoloration; 2 – Mild sclerosis: slightly more yellow or whitish dentin with up to 50% discoloration, intermediate between grades 1 and 3; 3 – Evident sclerosis: darker yellow or whitish dentin with more than 50% discoloration, between grades 2 and 4; 4 – Significant sclerosis: dentin with a glassy appearance, dark yellow or brown coloration, and high translucency or transparency.

Marginal tissue recession was classified as follows^28^: I – Recession not extending to the mucogingival junction; II – Recession extending to or beyond the mucogingival junction without interproximal bone loss; III – Involvement of the mucogingival junction and interproximal bone crest, possibly affecting tooth position; IV – Advanced mucogingival involvement with severe horizontal or interproximal bone loss.

Further details on these procedures can be found in our previous paper.^47^

### Randomization

Each group was identified by a number placed in a sealed envelope, which was used by the operator to randomly assign the restorative procedure to each NCCL. Each patient received restorations using all four types of adhesion strategies.

### Restorative Procedures

Two operators (postgraduate students) performed the restorative procedures. A prophylaxis with pumice and water was completed prior to restoration. The shade was selected, initial photographs were taken, and local anesthesia was administered when necessary. Relative isolation was achieved using cotton rolls and suction. The NCCLs were not subjected to any type of cavity preparation.

For the SBU group, the adhesive system was applied to the NCCLs with agitation for 20 s on a slightly dry surface. A gentle air stream was then applied for 5 s, and the adhesive was light-cured for 10 s (Radii-cal, 1200 mW/cm², SDI, Victoria, Australia).

For the E-SBU group, conditioning with 37% phosphoric acid (Total Etch – Ivoclar Vivadent, Liechtenstein) was applied to the enamel margins for 15 s, followed by rinsing with water for 20 s and gentle drying. The universal adhesive was then applied as described for the SBU group.

Both SBU and E-SBU groups were restored using a nanoparticulate composite resin following the incremental technique. Each increment was light-cured for 20 s, with the final increment cured for 40 s. Finishing and polishing were performed using fine and extra-fine diamond burs, followed by sequential polishing discs (Sof-Lex Pop-On, 3M ESPE, St. Paul, USA).

For the RMGIC group, Vitremer was used according to the manufacturer’s instructions. After prophylaxis, the surface was rinsed and dried, and the primer was applied with a brush and light-cured for 20 s.

For the E-RMGIC group, 0.1 M EDTA was applied with a brush for 60 s, followed by a 30-s rinse and dry. Restorations were performed without primer application to allow chemical bonding between the cement and dentin.^11^

The glass-ionomer cement was handled using a powder-to-liquid ratio of 1:1 by volume (one portion of powder to one drop of liquid) on a glass slab with a plastic spatula for approximately 45 s. After mixing, the material was inserted into the cavity using a Centrix-type syringe with disposable tips. The restorative material was condensed against the cavity walls; no matrix system was used during this procedure. The resin-modified glass-ionomer cement was light-cured for 40 s. Finishing and polishing were performed in the same manner as for the composite resin restorations. Finally, a thin layer of surface protector (Finishing Gloss, 3M ESPE, St. Paul, USA) was applied and light-cured for 20 s.

All materials‘ qualities can be seen in the previous paper.^47^

### Clinical Evaluation

The evaluations were performed through direct clinical observation using visual and tactile inspection with a flat mouth mirror, a periodontal probe, and illumination from a dental reflector. The criteria for classifying the restorations were based on those established by the United States Public Health Service (USPHS). Alpha and Bravo scores were considered successful outcomes, while Charlie scores were classified as failures.^47^ Two calibrated evaluators (different from operators), blinded to the group assignments, performed the evaluations and assigned scores. In cases of disagreement, a consensus was reached through discussion. DMFT, VPI, and GBI indexes, as well as restorations, were evaluated at baseline and at 1, 2, 3, 4, and 5 years.

### Statistical Analysis

The Kappa test was used to analyze inter-evaluator agreement. The Wilcoxon Signed-Rank test was applied to compare the four groups with respect to DMFT, VPI, and GBI indexes. Survival estimates for restoration longevity were assessed using the Kaplan–Meier method, considering all evaluation criteria. The equality test of two proportions was used to compare the four groups at each evaluation, as well as within each group over time, according to the USPHS criteria. Annual failure rates were also calculated.

Multiple logistic regression analysis was conducted to examine the influence of various factors (pain perception, cavity dimensions, tooth type, degree of sclerosis, internal angles, and degree of marginal tissue recession) on restoration survival, with retention as the dependent variable.

No intention-to-treat analysis was performed. All statistical analyses were performed using SPSS version 20 and SigmaPlot version 13. A significance level of 5% was adopted.

## RESULTS

In total, 50 patients (34 male and 16 female) with a mean age of 61 years (range, 38–92 years) participated in the present study. A total of 200 restorations were performed with a homogenous distribution per group in terms of NCCL characteristics.^47^

**Fig 1:**
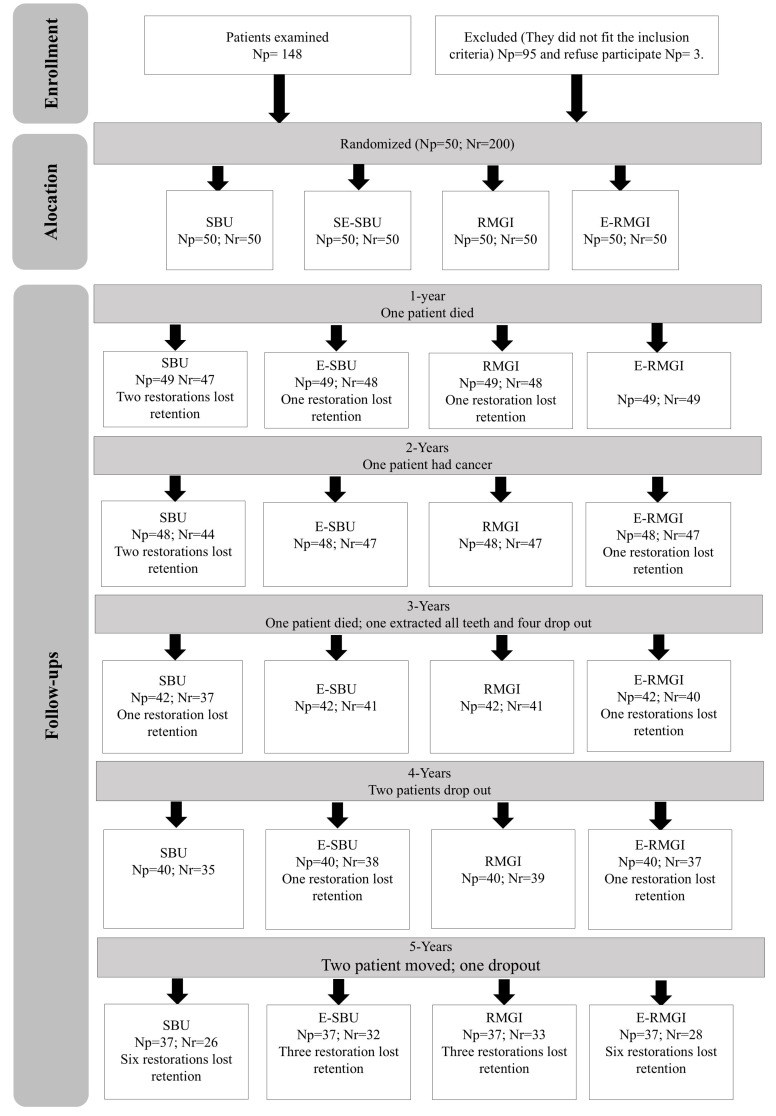
Consort flowchart.SBU: adhesive restorative system; E-SBU: adhesive restorative system with selective enamel conditioning; RMGIC: resin-modified glass-ionomer cement; E-RMGIC: resin-modified glass-ionomer cement with pretreatment with ethylenediaminetetraacetic acid; Np: number of patients; Nr: number of restorations.

After 5 years, 37 patients (74%) returned for evaluation. The number of patient dropouts and restorations assessed at each follow-up is shown in Figure 1. The reasons for loss to follow-up were described according to the Consolidated Standards of Reporting Trials (CONSORT).^48^

The Kappa test showed excellent inter-examiner reproducibility (κ = 0.993). Regarding DMFT, there was a gradual and statistically significant increase over time, with a significant difference between baseline (17.5 ± 4.8) and 5 years (21.5 ± 3.7) (P ≤ 0.05). No significant difference was found for VPI between baseline (41.4 ± 24.3) and 5 years (33.9 ± 19.2) (P ≥ 0.05). However, there was a significant reduction in GBI from baseline (14.1 ± 17.2) to 5 years (3.4 ± 9.0) (P ≤ 0.05).

**Fig 2:**
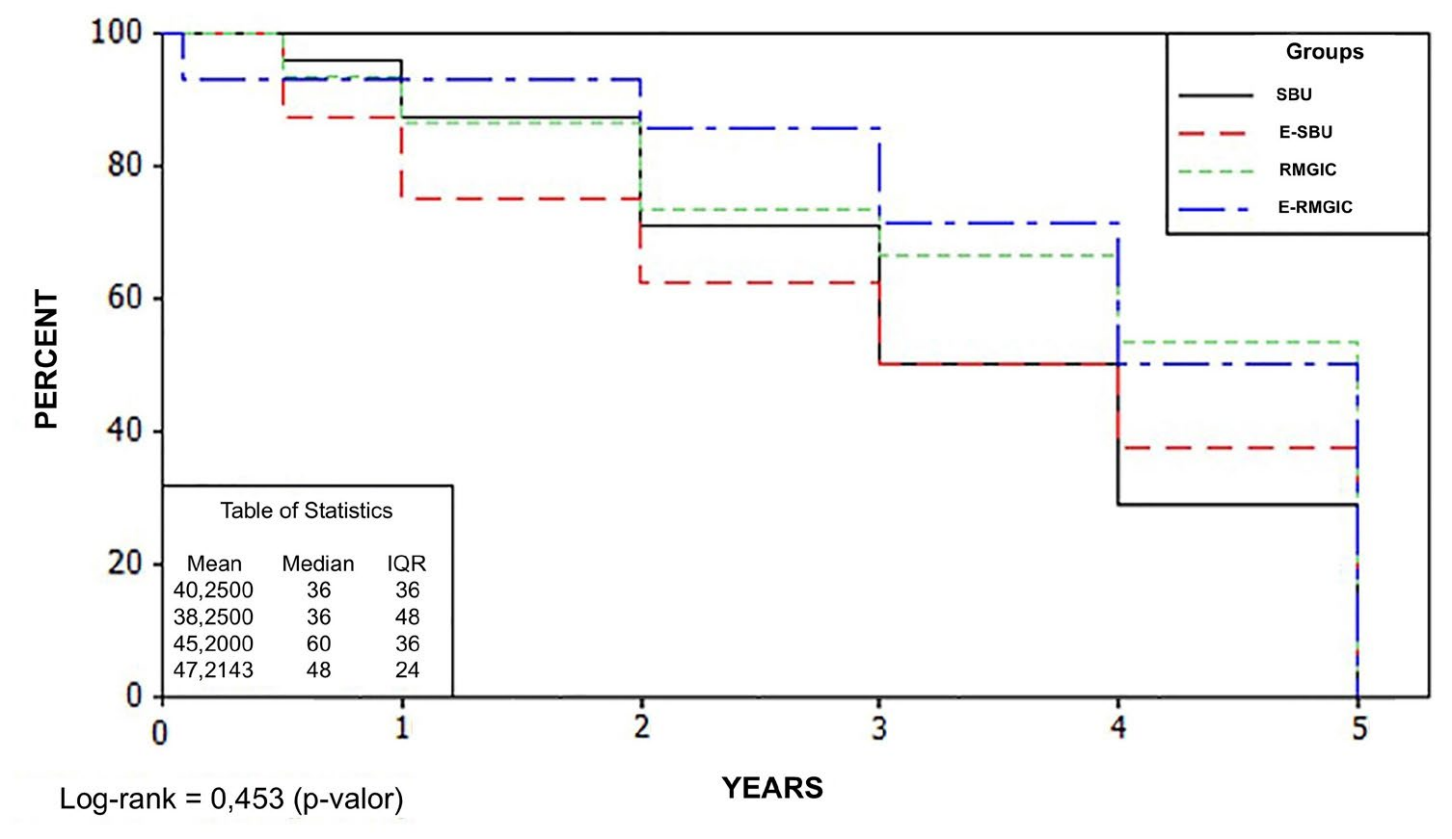
Survival curve of the four groups evaluated.

Survival analysis is presented in Figure 2, showing no statistically significant differences among groups (P = 0.453). The annual failure rates (%) over 5 years were 4.1, 1.7, 5.6, and 4.1 for the SBU, E-SBU, RMGIC, and E-RMGIC groups, respectively.

Clinical performance data are presented in Table 1. Statistically significant differences were observed among the four groups in terms of marginal integrity, marginal discoloration, surface texture, and wear (P ≤ 0.05). At the 5-year follow-up, a higher frequency of Bravo scores was recorded for the E-SBU group compared to the RMGIC and E-RMGIC groups for marginal integrity and compared only to the RMGIC group for marginal discoloration. Both groups restored with glass-ionomer cement exhibited higher Bravo scores for surface texture than those restored with composite resin, with significantly greater wear observed in the RMGIC group compared to E-SBU.

**Table 1:** Recall rates at each follow-up period and results in numbers of restorations per group according to each criterion at baseline, and 1, 2, 3, 4, and 5 years after the procedure

Periods	Groups	Recall rates	Score	Retention	Marginal integrity	Marginal discoloration	Surface texture	Wear	Secondary caries	Anatomical form	Surface staining	Color	Gingival tissue
**Baseline**	**SBU**	**100%**	**A/B**	50/0	49/1	50/0	50/0	50/0	50/0	49/1	50/0	36/14	47/3
**C**	0	0	0	0	0	0	0	0	0	0
**E-SBU**	**A/B**	50/0	49/1	50/0	50/0	50/0	50/0	50/0	50/0	41/9	47/3
**C**	0	0	0	0	0	0	0	0	0	0
**RMGIC**	**A/B**	50/0	50/0	50/0	49/1	50/0	50/0	47/3	50/0	37/13	47/3
**C**	0	0	0	0	0	0	0	0	0	0
**E-RMGIC**	**A/B**	50/0	48/2	50/0	50/0	50/0	50/0	48/2	50/0	39/11	44/6
**C**	0	0	0	0	0	0	0	0	0	0
**1-year**	**SBU**	**98%**	**A/B**	47/0	38/9	41/6	45/2	47/0	47/0	46/1	45/2	36/11	46/1
**C**	2	0	0	0	0	0	0	0	0	0
**E-SBU**	**A/B**	48/0	43/5	43/5	43/5	48/0	48/0	48/0	47/1	38/10	48/0
**C**	1	0	0	0	0	0	0	0	0	0
**RMGIC**	**A/B**	48/0	44/4	45/3	47/1	48/0	48/0	46/2	48/0	33/15	48/0
**C**	1	0	0	0	0	0	0	0	0	0
**E-RMGIC**	**A/B**	49/0	44/5	48/1	47/2	49/0	49/0	46/3	49/0	38/11	49/0
**C**	0	0	0	0	0	0	0	0	0	0
**2-years**	**SBU**	**96%**	**A/B**	44/0	35/9	35/9	43/1	44/0	44/0	43/1	43/1	36/8	43/1
**C**	4	0	0	0	0	0	0	0	0	0
**E-SBU**	**A/B**	47/0	40/7	37/10	43/4	46/1	47/0	47/0	45/2	38/9	47/0
**C**	1	0	0	0	0	0	0	0	0	0
**RMGIC**	**A/B**	47/0	38/8	42/5	43/4	46/1	47/0	45/2	46/1	33/14	47/0
**C**	1	1	0	0	0	0	0	0	0	0
**E-RMGIC**	**A/B**	47/0	37/10	41/6	42/5	47/0	47/0	44/3	47/0	35/12	47/0
**C**	1	0	0	0	0	0	0	0	0	0
**3-years**	**SBU**	**84%**	**A/B**	37/0	25/12	25/12	35/2	36/1	37/0	36/1	35/2	29/8	36/1
**C**	5	0	0	0	0	0	0	0	0	0
**E-SBU**	**A/B**	41/0	30/11	25/16	36/5	38/3	41/0	41/0	39/2	32/9	41/0
**C**	1	0	0	0	0	0	0	0	0	0
**RMGIC**	**A/B**	42/0	30/11	29/13	34/8	39/3	42/0	40/2	40/2	27/15	42/0
**C**	1	1	0	0	0	0	0	0	0	0
**E-RMGIC**	**A/B**	40/0	30/10	30/10	32/8	38/2	40/0	38/2	40/0	32/8	40/0
**C**	2	0	0	0	0	0	0	0	0	0
**4-years**	**SBU**	**80%**	**A/B**	35/0	15/20	20/15	33/2	33/2	35/0	34/1	32/3	27/8	34/1
**C**	5	0	0	0	0	0	0	0	0	0
**E-SBU**	**A/B**	39/0	16/23	18/21	31/8	34/5	39/0	37/2	36/3	30/9	39/0
**C**	1	0	0	0	0	0	0	0	0	0
**RMGIC**	**A/B**	38/0	23/15	22/16	19/19	29/8	38/0	33/5	36/2	26/12	38/0
**C**	1	0	0	0	1	0	0	0	0	0
**E-RMGIC**	**A/B**	37/0	23/14	27/10	14/23	31/6	37/0	35/2	36/1	30/7	37/0
**C**	3	0	0	0	0	0	0	0	0	0
**5-years**	**SBU**	**74%**	**A/B**	31/0	11/19	15/16	28/3	26/5	31/0	29/2	30/1	24/7	30/1
**C**	6	1	0	0	0	0	0	0	0	0
**E-SBU**	**A/B**	34/0	12/22	12/22	26/8	29/5	34/0	31/3	29/5	23/11	34/0
**C**	3	0	0	0	0	0	0	0	0	0
**RMGIC**	**A/B**	32/0	17/12	20/12	12/20	21/11	32/0	27/5	29/3	21/11	32/0
**C**	4	3	0	0	1	1	0	0	0	0
**E-RMGIC**	**A/B**	31/0	18/12	18/13	7/24	22/9	31/0	27/4	30/1	25/6	31/0
**C**	6	1	0	0	0	0	0	0	0	0
Alpha and Bravo scores were considered successful outcomes, while Charlie score was classified as failure.^47^

**Fig 3a to f:**
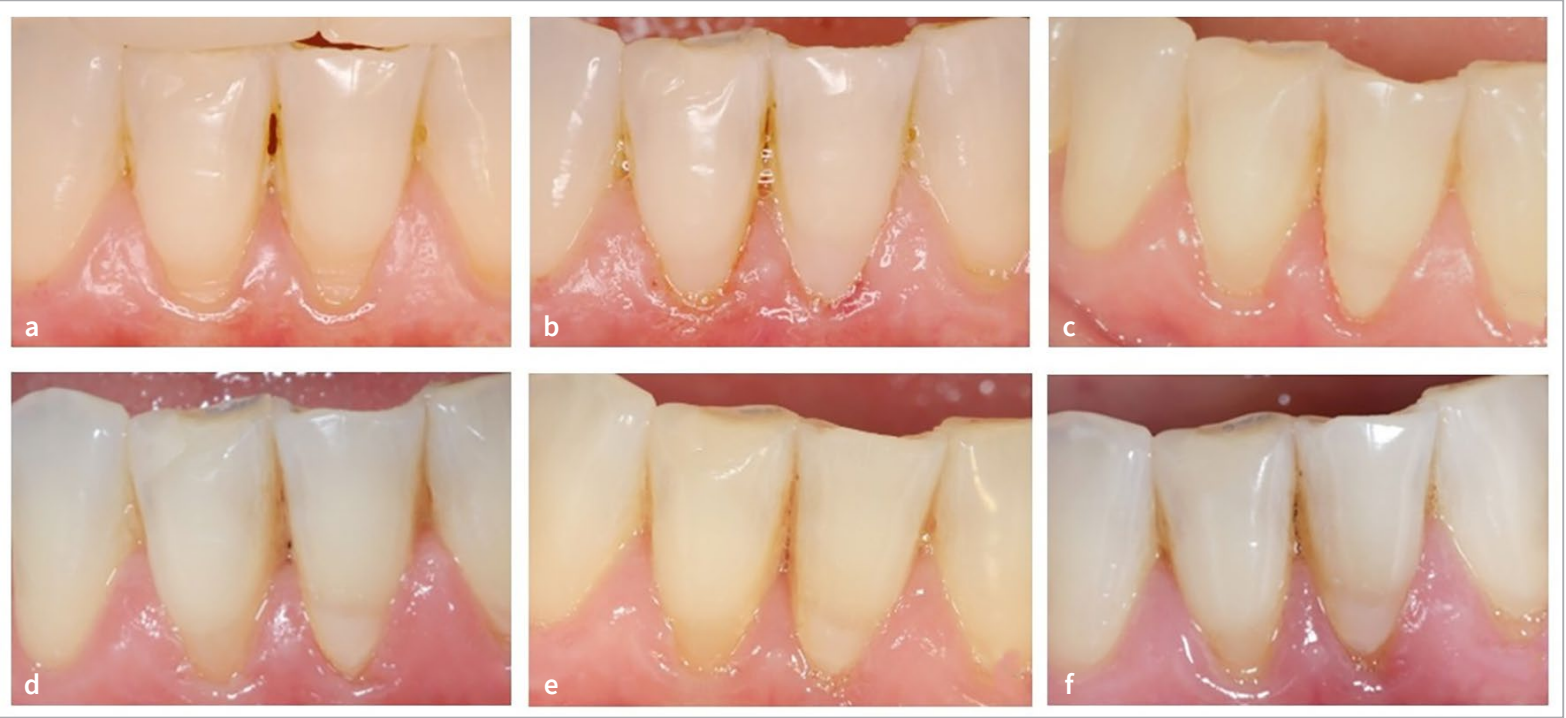
Clinical images of NCCLs restorations. (a) Preoperative image of mandibular right and left central incisors with NCCLs; (b) Baseline images of teeth from each group: mandibular left central incisor – SBU, mandibular right central incisor – RMGIC; (c) One-year follow-up showing an Alpha score for all evaluated criteria, except the mandibular left central incisor, which received a Bravo score for soft tissue health (mild inflammation); (d) Two-year follow-up with maintenance of previous scores and Bravo score for color (subtle visualization between tooth and restoration) in both teeth; (e) Four-year follow-up showing Bravo score for surface texture (slightly rough) in the mandibular right central incisor, and for color (subtle visualization between tooth and restoration) in both teeth; other criteria were considered as Alpha scores; (f) Five-year follow-up with maintenance of previous scores. Progression of both NCCLs was also noted, but the criteria used do not include this change.

**Fig 4a to f:**
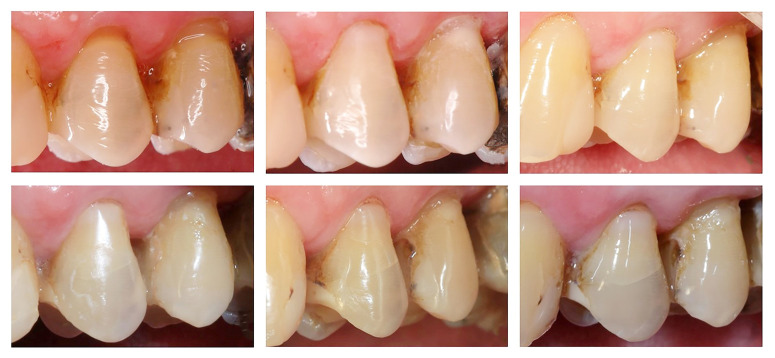
Clinical images of the NCCLs restorations. (a) Preoperative image of maxillary left first pre-molar and maxillary left second pre-molar with NCCLs; (b) Baseline image of teeth from each group: maxillary left first pre-molar – E-SBU, maxillary left second pre-molar – E-RMGIC,which received Bravo score for anatomical form (the restoration has a slight over-contour); (c) One-year follow-up showing Alpha scores (considered successful outcome) for all criteria, except for maxillary left second premolar, which received Bravo scores for marginal integrity (visual evidence of marginal discoloration at de tooth/restoration interface, which can be removed with polishing), anatomical form (the restoration has a slightly sub-contour), and color (subtle visualization between tooth and restoration); (d) Two-year follow-up with maintenance of previous scores; (e) Four-year follow-up showing a Bravo score for marginal integrity (visual evidence of marginal discoloration at the tooth/restoration interface, which can be removed with polishing) in the maxillary left first premolar, and Bravo scores for marginal integrity (visual evidence of marginal discoloration at the tooth/restoration interface, which can be removed with polishing), anatomical form (the restoration has a slightly sub-contour), color (subtle visualization between tooth and restoration), and surface texture (slightly rough) in the maxillary left second premolar; (f) Five-year follow-up with maintenance of previous scores.

No statistically significant differences were found for color match or the incidence of secondary caries over time (P ≥ 0.05). Representative clinical images of the restorations from all groups are shown in Figures 3 and 4.

Multiple logistic regression analysis (Table 2) revealed that restoration retention was significantly influenced by the type of tooth (premolars; P = 0.01), the degree of dentin sclerosis (P = 0.02), and the degree of marginal tissue recession (P = 0.02).

**Table 2:** Influence of initial NCCLs characteristics on the restorations’ retention after 5 years (multivariate logistic regression)

	OR	95% CI	P value
Tooth type (premolar)	25.38	1.77–363.3	0.019
Degree of sclerosis	0.29	0.10–0.83	0.020
Degree of marginal tissue recession	0.15	0.03–0.73	0.019
OR: odds ratio; CI: confidence interval

## DISCUSSION

NCCLs should ideally be managed through conservative, preventive interventions. However, restorative treatment often becomes necessary due to factors such as lesion progression, dentin hypersensitivity, compromised esthetics, food impaction, and a negative impact on the patient’s quality of life.^16^ Composite resins and glass-ionomer cements are commonly employed to restore these lesions.^16^

*In-vitro* and *in-situ* studies have contributed significantly to the literature about NCCLs restorations; however, there is difficulty in finding response variables that can be reliably employed in the clinical situation.^5^ For instance, saliva is a critical biological factor that not only influences the progression of dental erosion but can also affect the longevity and performance of NCCL restorations.^5^ Additionally, the dentin substrate present in NCCLs *in vivo* exhibits significant structural and morphological differences compared to the standardized substrates typically used in laboratory settings.^6^

Long-term clinical trials are essential for understanding the effectiveness and durability of restorative materials over extended periods. However, for such studies to yield reliable outcomes, maintaining a low dropout rate is critical, as higher attrition can significantly impact data interpretation and statistical validity.^2^ This limitation can be mitigated through a split-mouth study design, as employed in the present research. In this study, a dropout rate of 26% was observed, which aligns with previous clinical trials on NCCL restorations with follow-up periods of 5 years or more, where dropout rates ranged from 12% to 44%.^13,26,31,40,44^

Although the literature includes several recent clinical trials with follow-ups of 5 years or longer assessing the performance of universal adhesive systems in NCCLs,^13,26,30,31,40,44^ there is a relative scarcity of long-term studies evaluating resin-modified glass-ionomer cement restorations in these lesions.^10,12,56^ Furthermore, the use of EDTA as a pretreatment for NCCLs restored with glass-ionomer cement has been infrequently studied.^14,15,33,47^ In this context, this discussion compares the results presented in clinical trials of long-term follow-ups with those of the present study.

This clinical trial incorporated the evaluation of specific clinical indexes for each patient to better assess their overall oral condition. A statistically significant improvement in the GBI was observed over time, which can be attributed to patient education on oral hygiene and the provision of brushing kits during follow-up appointments. A recent study evaluating the impact of periodontal health education on oral hygiene motivation in patients with gingivitis found that even a single educational session was sufficient to encourage adherence to oral care routines.^17^ Additionally, the increase in the DMFT index over the study period reflects the ethical commitment to provide participants with all necessary dental treatments throughout the trial.

The analysis of adhesive interfaces *in vivo* reveals important characteristics of the degraded interface that helps to understand which aspects are determinant to failure.^5,6^ In this context, survival rate is a critical metric for predicting the long-term performance of restorative materials in NCCLs. The present study found no significant differences among the four adhesion strategies after 5 years, supporting the acceptance of the first null hypothesis. It is important to note that all restorations classified as Charlie under any USPHS criterion were considered failures in the survival analysis. The cumulative survival rates observed were 52% for SBU, 64% for E-SBU, 66% for RMGIC, and 56% for E-RMGIC, with no statistically significant differences among the survival curves (Fig 2). Previous studies assessing NCCLs restored with composite resins over similar follow-up periods have reported higher survival rates for selective enamel etching compared to the self-etching approach,^13,26,30^ which aligns with the results of the present study. Furthermore, other long-term clinical trials comparing composite resins and glass-ionomer cements have demonstrated superior outcomes for glass-ionomer restorations after 5 and 7 years.^10,12^

NCCLs are characterized by hypermineralized dentin and denatured collagen, which present a more challenging environment for adhesion.^5,39^ Following the discussion of USPHS parameters, retention is a key parameter in assessing the longevity of restorative materials. A systematic review reported that glass-ionomer cements exhibited significantly better clinical performance than composite resins in terms of retention, while other clinical parameters showed comparable outcomes between the two materials.^3^ In the present study, statistically significant differences among the groups were observed for marginal integrity, marginal discoloration, surface texture, and wear, leading to the rejection of the second null hypothesis.

In the SBU group, six restorations were lost; in contrast, only three restorations lost retention in the E-SBU group, after 5 years. It is important to emphasize that earlier debonding occurred in the SBU group. These findings align with previous clinical trials, which have reported superior retention performance for the same universal adhesive system when applied using the selective enamel etching technique compared to the self-etch mode over a 5-year follow-up.^13,26^ Similar outcomes have been reported with other universal adhesive systems, showing improved retention rates in the selective enamel etch mode after 5 and 6 years.^30,31^ Supporting these clinical observations, a systematic review and meta-analysis concluded that selective enamel etching prior to the application of self-etch adhesives in NCCLs significantly enhances the retention of composite restorations.^51^

Also concerning the retention criterion, although the E-RMGIC group exhibited a similar number of retention losses as the SBU group after 5 years, it demonstrated fewer retention failures than the SBU group until the 4-year follow-up.^47^ The glass-ionomer used in this study (Vitremer) has two adhesion mechanisms: first, based on its auto-adhesive capacity, establishing ionic interactions between carboxyl groups of polyalkenoic acid and hydroxyapatite in tooth structure; and second, micromechanical interlocking of the polymer.^10,12^ Despite this, there is a lack of clinical trials investigating the use of EDTA as a pretreatment for ionomeric restorations in NCCLs, limiting direct comparison with the present findings. EDTA is recognized as a clinically relevant MMPs inhibitor, potentially serving as a viable alternative pretreatment in NCCLs, as it interacts with calcium and zinc ions that are essential for MMPs activity.^53^ An *in-vitro* study has demonstrated that EDTA pretreatment can enhance the bond strength of glass-ionomer cement to dentin.^11^ These findings may explain the complete retention of restorations in the E-RMGIC group during the first year, whereas all other groups exhibited at least one failure in the same period. However, it can be hypothesized that the benefits of EDTA pretreatment may diminish over time, leading to similar long-term retention performance compared to other adhesion strategies.

In this context, the effects of EDTA may have contributed to the lower incidence of Bravo scores for marginal integrity observed in the E-RMGIC group. After the 5-year follow-up, the highest percentage of Bravo scores was found in the composite resin groups, which showed statistically significant differences compared to both RMGIC and E-RMGIC groups. The adhesive system used in this study contains HEMA, which enhances resin monomer penetration into demineralized dentin; however, HEMA is also known for promoting water sorption and the formation of poly-HEMA hydrogels, making the adhesive interface more susceptible to hydrolytic degradation over time.^13^ Long-term clinical trials have consistently reported fewer marginal defects in NCCLs restored with glass-ionomer cements compared to composite resins.^10,12^ Additionally, an in-situ study identified an enamel-like layer forming adjacent to ionomer restorations, indicating a potential “mineralizing” effect that may enhance marginal integrity.^57^ Notably, the E-SBU group was the only one that did not exhibit any Charlie scores for marginal integrity. A previous 5-year clinical study using the same universal adhesive system reported better marginal adaptation outcomes with the selective enamel etch mode versus the self-etching mode.^13^ Although Ruchel et al (2023) observed a greater number of Bravo scores with the etch-and-rinse mode compared to the self-etch mode after a similar follow-up period, no statistically significant difference was found, similar to the present study.

Regarding marginal discoloration, both groups restored with ionomer-based materials exhibited a higher number of Bravo scores compared to those restored with composite resins after 5 years; however, this difference was not statistically significant. These results corroborate previous studies in which no Charlie scores were reported for either ionomer or composite resin restorations in NCCLs with respect to marginal discoloration after 5 and 7 years of follow-up.^10,12^ Furthermore, no significant difference was found between the two composite resin groups (SBU and E-SBU) in the present study, which is consistent with findings from prior research reporting 17.2% of restorations with selective enamel etching and 45.8% with self-etching presented minor marginal discoloration after 6 years.^26,30,31,44^ However, our results diverge from those findings, as the E-SBU group in the present study exhibited a higher number of Bravo scores than the SBU group. It is important to note that those earlier studies employed different universal adhesive systems, which may explain the discrepancy. Additionally, in a separate 5-year clinical study, marginal discoloration was found to be significantly influenced by the adhesive strategy employed – specifically between etch-and-rinse (2-ER) and self-etch (1-SE) modes.^13^

In relation to surface texture, both RMGIC and E-RMGIC groups presented a statistically higher percentage of Bravo scores compared to the groups restored with composite resin after 5 years. Additionally, a significantly greater percentage of wear was observed in the RMGIC group (34.4%) compared to the E-SBU group (14.7%) at the same period of evaluation. Most long-term clinical studies assessing the performance of NCCL restorations using composite resin or glass-ionomer cements have not included surface texture and wear among their evaluation criteria, which makes direct comparisons with the present study challenging. This omission is likely due to the inherent differences in the surface characteristics of these materials, which may lead researchers to exclude these parameters. Nonetheless, given the distinct surface properties and mechanical behavior of composite resins and glass-ionomer cements, it is reasonable to expect significant differences in these criteria.^4^

The present clinical trial revealed significant differences between baseline and the 5-year follow-up for all parameters, except for color and secondary caries, rejecting the third null hypothesis. The absence of significant changes in secondary caries was expected, as the treated lesions were not of carious nature.^5^ In fact, other studies on NCCL restorations have also reported no significant differences in secondary caries between baseline and long-term follow-ups.^10,12,13^ Composite resins generally maintain satisfactory color stability in the long term, although variations may occur depending on the specific material used.^35,49^ Conversely, resin-modified glass-ionomer cement typically exhibit lower color stability than composite resins.^49^ However, in the present study, both ionomer-based groups demonstrated favorable color stability after 5 years. In this context, the longevity of restoration color is significantly influenced by factors such as proper photopolymerization and adequate finishing and polishing.^3^ Moreover, a notable decrease in retention was observed for the self-etching strategy between baseline and 5-year follow-up in a clinical trial.^13^ Several clinical studies on NCCL restorations have not compared baseline and recall data, which made it difficult to discuss this subject.^26,30,31,44^

The fourth null hypothesis was rejected, as the logistic regression analysis revealed that the location of the restoration in premolars, the degree of dentin sclerosis, and marginal tissue recession significantly influenced restoration retention. Of the 200 restorations performed, 125 were placed in premolars,^47^ and among the 19 restorations that failed due to loss of retention over the 5-year period, 11 occurred in premolars. NCCLs are most frequently observed in premolars and have been strongly associated with factors such as aging and dental erosion.^7,19,52,58^ The elevated prevalence and increased rate of retention loss in premolars may be attributed to the greater occlusal loading and the higher incidence of premature occlusal contacts characteristic of these teeth.^58,61^

Furthermore, only two failed restorations were associated with dentin classified as degree 4 sclerosis, while the remaining failures were similarly distributed across degrees 1 (n = 6), 2 (n = 5), and 3 (n = 6). Sclerotic dentin differs significantly from sound dentin in terms of its response to acid etching and is more frequently observed in older individuals.^16^ The hypermineralized surface often found in these cervical lesions is resistant to demineralization, reducing adhesive effectiveness. Consequently, it is recommended to mechanically roughen the lesion surface using a fine-grit diamond bur and to extend the etching time to 30 s in order to enhance bonding and retention.^16^ Additionally, 68% of NCCL restorations were graded as I for marginal tissue recession. While short-term studies have generally found no correlation between restoration performance and factors such as tooth type or dentin sclerosis,^20,47^ the current trial provides novel insight through logistic regression analysis. Although no long-term clinical studies of over 5 years have employed this method to investigate associations between initial lesion characteristics and restoration outcomes.^10,12,13,26,30,31,40,44,51,56^ However, the limited and inconclusive nature of existing clinical evidence highlights the need for further investigation.^18^

The effect of EDTA seems to be of short duration,^55^ primarily due to its high water solubility, which facilitates its complete elimination during rinsing procedures.^54^ As a result, the stability of the adhesive interface following EDTA pretreatment is likely attributed more to its mild demineralizing capacity than to inhibition of MMPs.^24^ Finally, in cases where esthetics is a primary concern, the combined use of resin-modified glass-ionomer cement and composite resins – commonly referred to as the sandwich technique – has emerged as a viable restorative strategy.^33^ This approach appears to mitigate restoration failure, as studies have reported higher retention rates for NCCL restorations using the sandwich technique compared to those restored solely with composite resin.^33^ Moreover, there remains a clear need for the continued development and clinical evaluation of therapeutic adhesive systems that offer bio-protective properties, such as inhibition of enzymatic degradation and promotion of remineralization.^8^

## CONCLUSION

After 5 years, the four different adhesion strategies demonstrated similar survival outcomes. However, more initial marginal defects were observed across all groups, with a higher incidence in the selective enamel etching groups compared to the ionomer-based strategies. Ionomer restorations exhibited a notable reduction in surface luster over time.

### Acknowledgments

This study was supported by the São Paulo Research Foundation – FAPESP (grant number 2014/07086-0). This study was also financed in part by the Coordination for the Improvement of Higher Education Personnel – Brazil (CAPES) – Finance Code 001.
